# Neurophysiological Processing of Rise Time in Children with Autism Spectrum Disorder

**DOI:** 10.3390/brainsci16101010

**Published:** 2026-09-23

**Authors:** Victoria Manasevich, Daria Kostanian, Olga Sysoeva

**Affiliations:** 1 Center for Research on Talent Development, Sirius University of Science and Technology, 354340 Sirius, Russia; kostanyan.dg@talantiuspeh.ru (D.K.);; 2 Laboratory of Human Higher Nervous Activity, Institute of Higher Nervous Activity and Neurophysiology, RAS, 117485 Moscow, Russia

**Keywords:** autism spectrum disorder, rise time, auditory processing, event-related potentials, EEG, P1, N2, children

## Abstract

**Highlights:**

**What are the main findings?**

- Children with ASD and TD peers showed distinct patterns of neural modulation across rise times, with P1 amplitude reduced in ASD at 15 and 60 ms (trend at 30 ms), indicating weaker encoding of rapid acoustic onsets.
- In ASD, N2 latency showed an atypical drop at 60 ms and P1N2 peak-to-peak amplitude reached its minimum at 60 ms, whereas TD children showed a systematic N2 latency increase and a gradual P1N2 decrease from 15 to 240 ms.

**What are the implications of the main findings?**

- Auditory ERPs to simple non-speech sounds can detect group differences in rise time processing, and the selectivity of this effect—present for rapid but not the longest rise times—highlights the importance of assessing a range of rise times in ASD.
- Atypical neural encoding of rapid acoustic onsets in ASD may contribute to speech and language difficulties.

**Abstract:**

Background: Amplitude rise time is a critical acoustic cue for speech perception, yet its neurophysiological processing remains uninvestigated in autism spectrum disorder characterized by communication deficits, sensory abnormalities and often speech delay. This study examined whether children with autism spectrum disorder differ from typically developing peers in auditory event-related potential components across a continuum of rise times. Methods: Electroencephalography was recorded during passive presentation of pure tones with five rise time values (15, 30, 60, 120, 240 ms) in 42 children (21 with autism spectrum disorder, 21 typically developing, aged 4–10 years). Linear mixed models were used to analyze latencies and amplitudes of P1 and N2 components. Results: Children with autism spectrum disorder showed a less systematic pattern of neural modulation across rise times compared to typically developing peers. P1 amplitude was reduced at 15 and 60 ms (trend at 30 ms), with no significant group differences at longer rise times. N2 latency showed an atypical decrease at 60 ms compared to 30 ms, contrasting with the systematic increase observed in typically developing children. P1N2 amplitude reached a minimum at 60 ms in autism spectrum disorder, whereas typically developing children showed a gradual decrease across rise times. Conclusions: These findings provide the first evidence of atypical neurophysiological processing of rise time in children with autism spectrum disorder, with the most pronounced differences in the range of rapid acoustic onsets. Group differences were not detected at longer rise times, suggesting a selective rather than global deficit in temporal envelope processing.

## 1. Introduction

Rise time (RT) is one of the most important acoustic characteristics of auditory stimuli, playing a critical role in the perception of speech and non-speech sounds. From a physical standpoint, amplitude rise time refers to the duration over which a sound’s amplitude increases from its onset to its maximum value. This temporal cue defines the onset abruptness of a sound, directly influencing its perceptual quality: shorter RTs create more percussive onsets, while longer RTs produce smoother, more gradual onsets [1,2].

The perceptual relevance of RT spans multiple levels of the speech hierarchy. Critically for the present study, RTs in the range of 15–60 ms are essential for distinguishing stop consonants from glides and affricates from fricatives [3,4], while longer RTs (120–240 ms) contribute to syllabic and prosodic structure [5,6]. This dual role makes RT a particularly informative parameter for investigating auditory processing across both segmental and suprasegmental levels—and, importantly, for identifying which level may be selectively affected in clinical populations.

At the neurophysiological level, RT processing is robustly reflected in auditory event-related potential (ERP). A recent systematic review of electroencephalographic (EEG) studies [7] provides the foundational synthesis for the present work, confirming that increasing RT systematically modulates key ERP components, generally reducing their amplitude and prolonging their latency. Neural sensitivity to RT varies across processing stages: brainstem responses encode microsecond differences, while cortical long-latency components like N1-P2 optimally discriminate changes in the tens-to-hundreds of milliseconds range. However, this review did not include studies in autism spectrum disorder (ASD), as no such studies existed at the time of the search. Thus, while the review provides a comprehensive synthesis of RT processing in typical development and dyslexia, the neurophysiological mechanisms underlying RT processing in ASD remain unexplored.

Key evidence for atypical RT processing in clinical populations comes from studies in developmental dyslexia. In particular, altered ERP morphologies, such as attenuated P1 amplitude modulation by RT [8], and specific patterns of mismatch negativity (MMN) in response to RT deviants [9,10] have been reported in children with dyslexia. In contrast, the neurophysiological mechanisms underlying RT processing in Autism Spectrum Disorder (ASD) remain unexplored. This is a critical oversight, as converging evidence indicates fundamental disruptions in early auditory processing in ASD, including deficits in temporal envelope resolution [11] and delayed development of phonological categories [12]. Given the central role of RT sensitivity across the speech hierarchy and the established auditory processing anomalies in ASD, investigating the neurophysiological correlates of RT processing in this population is a logical next step. Such research could determine whether atypical RT encoding represents a shared or disorder-specific mechanism underlying language and communication difficulties associated with ASD. Thus, the present study addresses a clear research gap: to date, no study has directly examined how children with ASD process amplitude rise time using auditory ERPs.

To address this gap, the present study aims to investigate the electrophysiological markers of amplitude RT processing (using a set of RTs: 15, 30, 60, 120, 240 ms) in children with ASD compared to typically developing (TD) peers, focusing on both the response magnitude (amplitude) and temporal dynamics (latency) of auditory ERPs. As cortical responses are predominantly generated in the auditory cortex and adjacent supratemporal areas, with a well-documented right-hemisphere bias for processing of temporal features such as RT, voice onset time and duration changes for both speech and non-speech sounds [13,14], we focus to lateral fronto-central electrodes to examine the laterality effects that may provide additional insight into the neural organization of RT processing, particularly in populations where auditory temporal processing is atypical. We implicated children aged 4–10 years as this period is characterized by the rapid development of language skills and maturation of the auditory cortex [15]. Moreover, these age ranges appeared to be a rather understudied period providing a need for more extensive research in this important period of child development. Children with ASD were compared to sex- and age-matched TD peers to control for known modulators of auditory processing, including age and sex, which are particularly relevant given the 4:1 male-to-female ratio in ASD.

The following research questions were formulated:

(1) Do children with ASD differ from TD peers in the amplitude and latency of auditory ERP components (P1, N2, P1N2) across a continuum of rise times (15–240 ms)?
(2) Are these differences selective for a specific range of rise times?
(3) Do the observed neurophysiological patterns suggest atypical temporal envelope processing in ASD rather than a global auditory deficit?
(4) Whether the ERPs modulation by rise time in ASD is compatible with the results previously seen in developmental dyslexia.

We hypothesized that group differences could potentially emerge at both shorter RTs, relevant for segmental phonology (e.g., stop-glide contrasts), and longer RTs, relevant for prosodic processing.

This study provides the first empirical investigation of RT processing in children with ASD using auditory ERPs, thereby establishing a new line of research in the EEG literature on autism and offering a candidate neurophysiological correlate of atypical temporal envelope processing in this population.

The paper is organized as follows. Section 2 describes the participants, experimental paradigm, EEG recording and preprocessing, ERP analysis, and statistical approach, thereby providing the methodological basis for addressing the first two research questions. Section 3 presents the results for P1 amplitude and latency, N2 amplitude and latency, and P1N2 amplitude, directly addressing the first two research questions. Section 4 discusses these findings in the context of auditory development, RT processing in ASD, and comparisons with other neurodevelopmental disorders, addressing the third and fourth research questions. Section 5 presents the conclusions, and Section 6 outlines the limitations of the study.

## 2. Materials and Methods

This section describes the participants, the experimental paradigm, the EEG recording and preprocessing pipeline, the ERP analysis, and the statistical approach used to address the research questions outlined in the Introduction.

### 2.1. Participants

The study involved two groups of participants: children with autism spectrum disorder (ASD) (n = 21, aged from 52 to 119 months, average age 83.5 (±18.2), 6 females) and their sex- and age-matched typical developing (TD) peers (n = 21, aged from 52 to 122 months, average age 83.8 (±19.1), 6 females). A total of 42 children (12 females), aged from 52 to 122 months, with an average age of 83.6 (±18.2), were included in the sample.

For clinical characterization of the sample, all participants underwent diagnostic assessment using internationally recognized gold-standard instruments. The ASD diagnosis was confirmed using the Autism Diagnostic Observation Schedule, Second Edition (ADOS-2) [16] administered by a certified specialist. In cases where the ADOS-2 results were inconclusive (n = 2), the Autism Diagnostic Interview-Revised (ADI-R) [17] was additionally employed to provide supplementary diagnostic information. These instruments together constitute the internationally recognized gold standard for autism diagnostic assessment [18]. All children in both groups were screened using the SRS-2 [19], a quantitative parent-report measure designed to assess autistic traits and social impairment across the general population. The ASD group showed elevated raw scores (mean = 82.5, SD = 24.0, range 31–137) and T-scores (mean = 67.9, SD = 10.1, range 46–90), consistent with clinically significant levels of autistic traits. While most children with ASD showed elevated SRS-2 scores in the clinical range (T ≥ 60), a subset (n = 3; ages 52–94 months, mean = 72.67 months) fell below this threshold. This is consistent with previous reports that some children with confirmed ASD may not show elevated SRS scores, particularly those with higher verbal abilities or less severe social-communication difficulties [20]. Thus, the SRS-2 was used as a quantitative measure of autistic traits rather than a diagnostic tool. In contrast, the TD group showed raw scores (mean = 34.7, SD = 13.8, range 21–65) and T-scores (mean = 48.9, SD = 5.1, range 42–59) within the typical range, indicating that autistic traits were within the expected range for the general population. SRS-2 scores for all participants are reported in Supplementary Table S1.

Objective measures of language ability and non-verbal cognitive level were available for a subset of participants. Language was assessed using the Preschool Language Scale, Fifth Edition (PLS-5) [21]. Standardized PLS-5 scores were available for 16 children with ASD (receptive: mean = 63.7, SD = 19.8, range 50–119; expressive: mean = 59.4, SD = 18.9, range 50–120; total language: mean = 60.6, SD = 19.1, range 50–121) and 14 TD children (receptive: mean = 96.8, SD = 18.8, range 71–133; expressive: mean = 95.1, SD = 23.9, range 50–145; total language: mean = 95.8, SD = 21.1, range 70–142). Non-verbal cognitive ability was assessed using the Leiter International Performance Scale, Third Edition (Leiter-3) [22]. Standardized Leiter-3 scores were available for 18 children with ASD (non-verbal IQ: mean = 93.0, SD = 12.6, range 74–115; non-verbal memory: mean = 91.1, SD = 26.1, range 48–133) and 18 TD children (non-verbal IQ: mean = 107.1, SD = 11.7, range 89–131; non-verbal memory: mean = 103.6, SD = 17.6, range 71–136). Notably, in the ASD group, four children did not complete the Leiter-3 non-verbal memory task due to their functional state. These measures were not included as covariates in the main statistical models because they were not available for the full sample; individual scores are reported in Supplementary Table S2.

Peripheral hearing status was confirmed through a parental report of normal hearing. Children with known or suspected hearing impairment were not included in the study. However, standardized audiological assessment was not performed for all participants, and this is acknowledged as a limitation.

### 2.2. Data Collection and Ethics

This research was carried out with the approval of the Sirius University Bioethics Committee (Bioethics Committee Opinion dated 13 July 2022) and in line with the principles of the Declaration of Helsinki. Prior to data collection, written informed consent was provided by the participants’ parents or guardians, and verbal assent was obtained from each child participant. All procedures for the collection and management of EEG and behavioral data followed the approved study protocols.

### 2.3. Stimuli and Procedure of EEG Experiment

Stimuli were presented through headphones, binaurally. While listening, participants watched cartoons of their choice without sound. Pure tones with five different RTs (15 ms, 30 ms, 60 ms, 120 ms, 240 ms) were used as stimuli. The tone frequency was 500 Hz, the duration was 400 ms, and the fall time was 50 ms. The order of stimulus presentation was quasi-random, with three constraints: (1) the total number of presentations of each stimulus type was equal to each other (150 presentations per stimulus); (2) the same stimulus was not repeated several times in a row; (3) two consecutive presentations of the same stimulus were separated by no more than 8 other stimuli. A total of three blocks with different sequences were presented. Each block included 250 trials, with 50 quasi-random presentations of each individual stimulus. The inter-stimulus interval was 450 or 550 ms, in random order.

### 2.4. EEG-Recording

Electroencephalographic (EEG) data were acquired in an electromagnetically shielded chamber. Recordings were obtained using a 32-channel (31—active, 1—referent) Brain Products actiCHamp system (Brain Products GmbH, Gilching, Germany) at a sampling rate of 50 kHz, in parallel with auditory stimulation. The signal was recorded without applying online filters (bandwidth range: DC to 10.3 kHz). The reference electrode was placed at the FCz site, and electrode impedances were maintained below 50 kΩ.

### 2.5. EEG Data Preprocessing

The preprocessing pipeline consisted of several steps implemented using the MNE-Python library v.1.9.0. First, a zero-phase bandpass FIR filter (0.5–40 Hz) was applied to all continuous EEG recordings to remove slow drifts and high-frequency noise. Next, visual inspection was performed to identify and mark bad channels; these channels were subsequently interpolated using spherical spline interpolation. To remove ocular artifacts, Independent Component Analysis (ICA) with 20 components was applied to each dataset. Components corresponding to eye blinks and saccades were identified based on their topographic distribution and time-course and were excluded from the signal. The data were then segmented into epochs from −100 ms to 500 ms relative to stimulus onset, with a baseline correction applied over the pre-stimulus interval (−100 to 0 ms). Epochs containing amplitude excursions exceeding ±250 µV were automatically rejected. The average number of epochs included in the analysis of each participant for each RT condition was 146.4 (±3.3, range 136–150) for the TD group and 139.0 (±10.1, range 109–150) for the ASD group. Finally, for each condition, evoked responses were computed by averaging epochs according to stimulus type per participant, and the EEG data were re-referenced to the average of the TP9 and TP10 mastoid electrodes to assess the maximum of brain activity of the auditory cortex and frontocentral channels [23,24].

### 2.6. ERP Data Analysis

For the analysis of event-related potentials, we performed a semi-automatic peak detection of the P1 and N2 components for each participant. These components were considered because they are predominant in the ERP configuration in this age group, whereas N1 and P2 are still immature [15].The analysis was guided by the topographical distribution of activity, which was the most prominent over fronto-central regions (Supplementary Figures S1 and S2), consistent with the maximal activation of the auditory cortex and adjacent supra-temporal areas. This fronto-central scalp distribution is characteristic of auditory processing and reflects the summation of activity from bilateral auditory cortices, aligning with the established generators of the P1 and N2 components [25,26]. To assess potential hemispheric differences (lateralisation), we focused our analysis on the lateral frontal electrodes FC5 (left-sided) and FC6 (right-sided) for each of the five RT conditions (15, 30, 60, 120, and 240 ms).

The time windows for peak detection were defined based on the grand-average waveforms across all conditions to ensure consistency. The target window for the P1 component was set to 65–200 ms, and for the N2 component to 200–450 ms post-stimulus onset [25,26].

Peak detection was implemented in a custom Python function. For each participant, electrode, and condition, the signal was inverted in polarity for the N2 component so that both P1 (positive) and N2 (after inversion) could be treated as positive deflections, allowing a unified search procedure. Within the predefined time window, local maxima were identified using scipy.signal.find_peaks with a minimum distance of 20 ms between peaks to avoid spurious high-frequency fluctuations. The highest peak within the window was selected as the component peak. For amplitude extraction, we computed the mean voltage within a ±10 ms window centred on the detected peak to reduce the influence of residual high-frequency noise [23]. For the N2 component, amplitude values were returned to their original negative polarity after peak detection.

Component latency was estimated separately using the 50% fractional area latency, defined as the time point at which 50% of the total absolute area under the curve within the search window was reached [24]. This measure is more robust for broad, slow components such as the N2 in children and is therefore reported instead of the peak latency. When the signal in the window lacked a positive deflection, the absolute (rectified) signal was used instead, and a fallback flag was recorded.

Finally, a peak-to-peak amplitude P1N2 was calculated as the difference between the P1 amplitude at its peak and the N2 amplitude at its peak (P1_amp_peak—N2_amp_peak). The final latency and amplitude values for the P1 and N2 components at electrodes FC5 and FC6 were extracted and stored for each participant and each RT condition. All detected components were visually verified on individual plots where the search windows were highlighted and the selected peaks were marked.

### 2.7. Statistical Analysis

In this section, we describe the linear mixed models and post hoc procedures used to test the effects of Group, RT, and Hemisphere. Statistical analysis was carried out using R version 4.5.2 (R Core Team, Vienna, Austria, 2025). To examine the effects on the neurophysiological parameters, we employed linear mixed models (LMM) fitted using the lme4 and lmerTest packages [27,28]. The models included Group (2 levels: ASD, TD), RT (5 levels: 15, 30, 60, 120, 240 ms), Hemisphere (2 levels: FC5, FC6), and all interactions as fixed effects, with Age as a continuous covariate. A random intercept for each participant ((1|ID)) was included to account for the nested structure of repeated measurements. A random-intercept-only structure was chosen because models with random slopes for RT by participant were unstable and did not converge. The random-intercept structure was therefore retained as the more reliable specification. Separate models were run for each dependent variable: P1 latency, P1 amplitude, N2 latency, N2 amplitude, and P1N2 peak-to-peak amplitude. Denominator degrees of freedom were estimated using the Satterthwaite approximation [27]. Linear mixed model results and pairwise post-hoc comparisons are reported in Supplementary Tables S3–S17.

Sensitivity analysis using Monte Carlo simulations was performed based on the fitted models. Fixed-effect estimates and variance components from the observed data were used to simulate new datasets, with the Group × RT interaction coefficients scaled across a range of values. Power was estimated as the proportion of simulations in which the Group × RT interaction reached significance at α = 0.05 (200 simulations per effect size). The simulation indicated that, with N = 42, α = 0.05, and 80% power, the minimum detectable effect for the Group × RT interaction corresponded to Cohen’s f ≈ 0.17 (partial η^2^ ≈ 0.027) across the three ERP components. When significant main effects or interactions were observed in the primary analysis, follow-up post hoc tests were conducted. To control for multiple comparisons, the Benjamini–Hochberg False Discovery Rate (FDR) procedure was applied separately to each family of post hoc tests (e.g., comparisons between RT levels and between groups).

### 2.8. Data Visualization

All figures were generated using two software environments. Event-related potential waveforms and topographic maps (Figure 1 and Supplementary Figures S1 and S2) were created using Python 3.10 with the MNE-Python library (v1.9.0). Violin plots showing group-level distributions and effects (Figure 2) were generated in R version 4.2.1 using the ggplot2 package (v3.5.1). Event-related potentials were plotted from the preprocessed and epoched data after baseline correction and artifact rejection, as described in Section 2.5. Group-level averages were computed by averaging individual participant waveforms within each group. Violin plots display the full distribution of individual participant values, with overlaid boxplots indicating the median and interquartile range. Statistical annotations (asterisks) were added manually based on the FDR-corrected *p*-values reported in the Results section. No generative AI tools were used in the creation, modification, or enhancement of the figures.

## 3. Results

This section presents the general ERP architecture and then the results for each ERP component in turn: P1 amplitude and latency, N2 amplitude and latency, and P1N2 amplitude. The grand-averaged ERPs in response to auditory stimuli with different RTs showed the expected pattern for this age, with predominant P1 and N2 components for both the ASD and TD groups. Figure 1 illustrates the morphology and waveform patterns across different RT conditions. Shading around each line represents the 95% confidence interval of the grand-averaged response. Vertical bars below the *x*-axis indicate the time windows used for peak detection: violet for P1 (65–200 ms) and green for N2 (200–450 ms).

Figure 2 summarizes the group differences in P1 amplitude (panel A), N2 latency (panel B), and P1N2 amplitude (panel C) across rise times. In all panels, asterisks indicate the level of statistical significance after FDR correction: * *p* < 0.05, ** *p* < 0.01, *** *p* < 0.001.

### 3.1. P1 Amplitude

The linear mixed model (LMM) revealed a significant main effect of Group (F (1, 42) = 6.63, *p* = 0.014, η^2^p = 0.136), with ASD demonstrating significantly lower P1 amplitude compared to TD, a significant main effect of Hemisphere (F (1, 378) = 7.28, *p* = 0.007, η^2^p = 0.019) with a larger P1 amplitude over the right-sided electrode FC6 (mean = 3.74 µV), compared to the left-sided FC5 (mean = 3.34 µV), and a significant main effect of RT (F (4, 378) = 2.42, *p* = 0.048, η^2^p = 0.025).

A significant Group × RT interaction was also observed (F (4, 378) = 3.29, *p* = 0.011, η^2^p = 0.034). Post hoc independent samples *t* tests across RT levels revealed lower P1 amplitude in ASD compared to TD at 15 ms (t (40) = −2.68, *p* (FDR) = 0.026) and at 60 ms (t (40) = −2.73, *p* (FDR) = 0.026), with a trend at 30 ms (t (40) = −2.09, *p* (raw) = 0.043, *p* (FDR) = 0.072). No significant group differences were observed at 120 ms (t (40) = −1.74, *p* (FDR) = 0.111) or 240 ms (t (40) = −0.85, *p* (FDR) = 0.400) (Table 1). Such a pattern of differences can be accounted for by an expected gradual decrease in TD children (e.g., 15 ms vs. 60 ms (t (20) = 2.26, *p* (raw) = 0.035), 15 vs. 120 ms (t (20) = 2.3, *p* (raw) = 0.032), 15 vs. 240 ms (t (20) = 2.26, *p* (raw) = 0.035), which, however, did not survive our strict FDR-correction rule (see Supplementary Materials). No such pattern was observed in children with ASD, whose P1 amplitudes were stable and low across RT conditions (Figure 2A).

### 3.2. P1 Latency

The LMM revealed significant main effects of RT (F (4, 378) = 24.79, *p* < 0.001, η^2^p = 0.208) and Hemisphere (F (1, 378) = 6.30, *p* = 0.013, η^2^p = 0.016) with longer P1 latency over the right FC6 (mean = 117.1 ms) compared to the left FC5 (mean = 113.7 ms).

Pairwise comparisons of RT levels across the whole sample (paired *t* tests, FDR corrected for 10 comparisons) showed that P1 latency at 120 ms was longer than at 15 ms (t (41) = −3.72, *p* (FDR) = 0.001), at 30 ms (t (41) = −4.71, *p* (FDR) < 0.001), and at 60 ms (t (41) = −2.73, *p* (FDR) = 0.013). P1 latency at 240 ms was longer than at 15 ms (t (41) = −5.93, *p* (FDR) < 0.001), at 30 ms (t (41) = −7.81, *p* (FDR) < 0.001), at 60 ms (t (41) = −6.01, *p* (FDR) < 0.001), and at 120 ms (t (41) = −3.06, *p* (FDR) = 0.006). No other comparisons were significant (all *p* (FDR) > 0.05).

### 3.3. N2 Amplitude

The LMM revealed a significant main effect of Hemisphere (F (1, 378) = 5.26, *p* = 0.022, η^2^p = 0.014) with larger N2 amplitude over the FC6 (mean = −5.96 µV) compared to the FC5 (mean = −5.53 µV) and a significant main effect of Age (F (1, 42) = 6.52, *p* = 0.014, η^2^p = 0.134).

Pearson correlation confirmed a significant negative relationship between age and mean N2 amplitude averaged across all RTs and electrodes (r = −0.366, *p* = 0.017), indicating increase in N2 amplitude with age.

### 3.4. N2 Latency

The LMM revealed a significant main effect of RT (F (4, 378) = 7.26, *p* < 0.001, η^2^p = 0.071) and a significant Group × RT interaction (F (4, 378) = 3.14, *p* = 0.015, η^2^p = 0.032).

Post hoc paired *t* tests across the whole sample showed that N2 latency at 240 ms was longer than at 15 ms (t (41) = −3.16, *p* (FDR) = 0.015), at 60 ms (t (41) = −3.81, *p* (FDR) = 0.005), and at 120 ms (t (41) = −2.73, *p* (FDR) = 0.031). Post hoc independent samples *t* tests across RT levels showed a trend to longer N2 latencies in ASD at 30 ms (t (40) = 2.40, *p* (raw) = 0.021, *p* (FDR) = 0.106). No other comparisons were significant (Table 2). Within group paired comparisons (paired *t* tests, FDR corrected for 10 comparisons per group) revealed distinct patterns. In the TD group, N2 latency at 240 ms was longer than for all other shorter RT, with the most pronounced difference that reached FDR-correction being with 30 ms (t (20) = −3.40, *p* (FDR) = 0.029). In the ASD group, N2 latency at 240 ms was also longer but only for one shorter RT of 30 ms (t (20) = −3.21, *p* (FDR) = 0.022). In addition to these previously reported latency increases for longer RT in TD, children with ASD demonstrated shorter N2 latency at 60 ms than at 30 ms (t (20) = 3.26, *p* (FDR) = 0.022), clearly contrasted with TD data.

### 3.5. P1N2

The LMM revealed significant main effects of RT (F (4, 378) = 4.23, *p* = 0.002, η^2^p = 0.043), Hemisphere (F (1, 378) = 23.31, *p* < 0.001, η^2^p = 0.058) with larger P1N2 amplitude over the FC6 (mean = 10.65 µV) compared to the FC5 (mean = 9.72 µV), and Age (F (1, 42) = 4.67, *p* = 0.036, η^2^p = 0.100), with P1N2 increasing with age, as well as a significant Group × RT interaction (F (4, 378) = 2.74, *p* = 0.029, η^2^p = 0.028).

Post hoc pairwise comparisons of RT levels across the whole sample (paired *t* tests) revealed that P1N2 amplitude at 60 ms (t (41) = 3.62, *p* (FDR) = 0.008), at 120 ms (t (41) = 3.41, *p* (FDR) = 0.008), and at 240 ms (t (41) = 2.91, *p* (FDR) = 0.019) were significantly lower than at 15 ms. Within-group paired comparisons (paired *t* tests, FDR corrected for 10 comparisons per group) for the Group × RT interaction revealed that in the TD group, P1N2 amplitude at 15 ms was higher than at 240 ms (t (20) = 3.81, *p* (FDR) = 0.011) and at 120 ms (t (20) = 3.49, *p* (FDR) = 0.012). In the ASD group, P1N2 amplitude at 60 ms was lower than at 15 ms (t (20) = 3.87, *p* (FDR) = 0.009) and at 30 ms (t (20) = 3.59, *p* (FDR) = 0.009). Post hoc group comparisons across RT levels revealed no significant differences (Table 3). No other comparisons were significant (all *p* > 0.05).

## 4. Discussion

This section discusses the findings in two parts. We first consider the general properties of RT processing in early childhood, and then turn to the atypical RT processing observed in children with ASD and its comparison to previous findings in developmental dyslexia, ending with potential implications of the observed neurophysiological abnormalities to speech and language processing. This study is the first to investigate the neurophysiological correlates of auditory RT processing in children with ASD compared to their TD peers. For this purpose ERPs to pure tones with varying RTs (15, 30, 60, 120, 240 ms) were recorded.

The main findings point to differences in how the auditory system responds across the RT continuum and processing stages in ASD compared to TD peers. While P1 latency modulation by RT, i.e., latency increases with increase in RT, was preserved in ASD, P1 amplitude did not show any sign of RT modulation, contrary to TD. Thus, the ASD atypicalities were observed starting from the earliest ERP component studied. In particular, ASD showed lower P1 responses at 15 and 60 ms (with a trend at 30 ms) compared to the TD group that might be triggered by the absence of P1 amplitude modulation in ASD, but not TD, who showed expected P1 amplitude decrease with increase in RT. For the N2 component, the RT modulated its latency, but differently for TD and ASD. In TD children, N2 latencies increased progressively with RT, while in ASD there was an atypical drop in latency for 30 vs. 60 ms RT. The P1N2 amplitude also showed a slightly atypical pattern of RT modulation compared to TD. Together, these findings suggest that the deficit in ASD is not limited to a specific RT but reflects a more general difference in how neural responses are modulated across the RT range, most evident for rapid acoustic onsets. We discuss these findings in detail below.

### 4.1. RT Processing in Early Childhood

Based on previous studies, investigating the neurophysiological processing of RT in children aged 4–10 years remains an underexplored area. Existing research in this age group has predominantly examined adult-like ERP components such as N1 and P2, as well as mismatch responses (MMN/MMR) in oddball paradigms (e.g., [8,10,29]). However, auditory cortex maturation in childhood is characterized by gradual changes in ERP morphology. In early and preschool years, the P1 and N2 components are more pronounced, while the N1 amplitude becomes clearly differentiated only by approximately 10–12 years of age [15]. This creates a methodological gap, as relying on “adult” markers (N1/P2) to assess RT processing in younger children may be suboptimal because these components are still immature and often poorly defined. Consequently, any investigation of auditory processing in this age range must consider both the developmental trajectory of ERP components and the potential modulating role of chronological age itself.

As expected, N1 and P2 components, which are the primary markers of RT effects in adults, were not yet well expressed in our pediatric sample, consistent with the known developmental trajectory of these components [28]. Other general age-related ERP changes were also confirmed in our study: N2 amplitude increased with age, consistent with reports that N2 amplitude grows during early and middle childhood, reaching a peak around 10–12 years of age [26,30]. The P1N2 measure also increased with age, consistent with the maturational trajectory of the underlying components. In contrast, no significant age-related changes were observed for P1 amplitude or latency, suggesting that early sensory registration (P1) undergoes its most pronounced changes earlier in childhood [31,32], whereas later integrative processes (N2) continue to develop through middle childhood [30]. Notably, all these developmental changes were not related to RT.

Another property of auditory processing that was observed in the present sample, irrespective of the diagnostic group, was the hemispheric asymmetry evident across multiple auditory responses: P1 amplitude, N2 amplitude, and the P1N2 measure were larger over the FC6 compared to the FC5, and P1 latency was longer over FC6. Crucially, these hemispheric differences were not modulated by RT; they were present across all RTs and did not differ between groups. This right-lateralized scalp pattern aligns with evidence that right-hemisphere auditory evoked responses discriminate temporal features of both speech and non-speech sounds [8], supporting the role of the right hemisphere in processing temporal sound characteristics. The absence of RT-dependent modulation of laterality suggests that hemispheric specialization in this context operates independently of the specific temporal structure of the stimulus, at least within the range of RTs tested.

Most important are patterns of RT modulation. In the present sample, the early cortical response (P1), reflecting basic sensory registration and sound detection [33,34,35], reliably encoded the temporal characteristics of stimulus onset. Consistent with previous findings in children [8], P1 latency increased with RT prolongation, with the longest latencies at 120 and 240 ms and shorter latencies at 15, 30, and 60 ms. P1 amplitude, in contrast, showed general amplitude decrease with RT prolongation, though within-group comparisons did not survive FDR correction, consistent with the previous findings in block designs [8,36]. In contrast to P1, where both amplitude and latency were modulated by RT, the N2 component, associated with higher-order auditory processes, including stimulus evaluation, discrimination, and categorization [37,38], showed RT sensitivity primarily in its latency. N2 latencies were longer at the longest RT (240 ms) compared to shorter RTs, which is consistent with the general sensitivity of N2 to RT variation reported in children of age 7–11 [8]. N2 amplitude did not vary systematically across RTs, indicating that the magnitude of the later-stage neural response was not modulated by RT in a simple manner, while the peak-to-peak P1N2 amplitude was lower at longer RTs (120 and 240 ms) compared to the shorter RTs (15, 30 ms), pointing to expected decrease with more gradual sound onsets (e.g., consistent with the earlier finding by Skinner and Jones (1968) [36], who reported a reduction in the P1N2 complex with RT increase in adults). This suggests that the sensitivity of the P1N2 measure to RT duration is preserved across development, despite differences in age and experimental design.

### 4.2. Altered RT Processing of Shorter RTs in ASD

While the general properties of auditory processing described above were largely preserved across the whole sample, the two groups diverged in how neural responses were modulated across RTs. In TD children, the pattern of modulation followed the expected direction with longer RTs resulting in longer latencies and smaller amplitudes of ERP components [7,8,36]. In ASD the pattern of modulation was different which we compared with that of TD and seen in other neurodevelopmental disorders below.

As our study is the first to investigate RT processing in ASD using auditory ERP components, it is impossible to compare it with previous neurophysiological reports on this population. Previous research on RT processing in clinical pediatric populations has primarily focused on dyslexia and implicated oddball design, where rare deviant stimuli interspersed with frequent standard. The results of these studies regarding mismatch response are highly inconsistent [9,27,39]. Regarding the main ERP components in children with dyslexia, Stefanics et al. (2011) reported that P1 amplitude decreased with RT prolongation, and both P1 and N2 showed prolonged latencies with longer RTs (90 ms) in this clinical group but only in the oddball condition [8]. At the same time, these ERP components in the block design did not differ between children with dyslexia and their TD peers for the RT considered (15 and 90 ms). This pattern clearly diverges from what is seen in our ASD sample. In particular, P1 amplitude was reduced at 15 and 60 ms (trend at 30 ms) compared to TD peers, indicating weaker encoding of rapid acoustic onsets in ASD. However, we should note that in our design stimuli with different RTs were presented within the same block with equal probability and quasirandomized order while in the study of Stefanics and colleagues, stimuli with different RTs were presented in different blocks. Our design might be more sensitive to the RT difference. At the same time, it is unlikely that children with dyslexia will show reduction in P1 when stimuli is intermixed as they showed an increase in P1 for the deviant stimuli in oddball block. Comparisons of other components to previous data obtained in clinical groups are limited by differences in RT and study design. Thus, it remains a direction for future research to examine, for example, whether the atypical N2 latency drop from 30 to 60 ms RT is specific to ASD or reflects broader aspects of the study design. Nonetheless, these findings suggest that the neural mechanisms underlying RT processing deficits in ASD are distinct from those observed in dyslexia, where deficits have been primarily associated with phonological processing and reading difficulties. In ASD, the observed atypicalities in amplitude modulation across RTs, particularly the non-monotonic trajectory of P1N2 and the atypical N2 latency pattern, point to more generalized disruptions in temporal envelope processing that may affect the encoding of dynamic acoustic features, potentially contributing to the broader language and communication difficulties characteristic of ASD.

The convergence of these atypical patterns at the 30–60 ms window suggests a broader difficulty in processing the acoustic boundary between rapid and gradual onsets, rather than a deficit limited to a single component. In speech perception, RTs in this range are critical for distinguishing phonetic contrasts such as stops versus glides (e.g., /ba/ vs. /wa/) and affricates versus fricatives (e.g., /tʃ/ vs. /ʃ/) [3,4]. In TD children, the progressive increase in N2 latency and the gradual decrease in P1N2 amplitude across RTs suggest a smooth neural differentiation between these categories. In ASD, however, this differentiation may be disrupted across multiple components. The present findings are therefore consistent with the hypothesis that atypical processing of rapid acoustic transitions may contribute to speech and language difficulties in ASD, although this link was not directly tested here and remains to be examined in future studies using speech stimuli.

One possibility is that the observed pattern of difficulties in the phonetically relevant window in ASD is related to the heterogeneity of the autism spectrum disorder, in which auditory processing profiles likely vary across individuals. In the present sample, children with ASD ranged from minimally verbal to fluent speech. Prosodic processing, which is associated with longer rise times, typically requires more developed language skills and can be most evident in verbally fluent individuals. Thus, the absence of group differences at longer RTs (120–240 ms) may reflect the wide range of language abilities in the ASD group, where subgroup-specific effects could be masked at the group level. Future studies with language-matched designs are needed to clarify whether longer RT processing is differentially affected in verbally fluent versus minimally verbal children with ASD.

Another mechanism that may contribute to the atypical pattern at shorter RTs is the rate of acoustic energy accumulation. Shorter RTs correspond to abrupt increases in sound intensity, placing high demands on early sensory registration. In ASD, such rapid energy onsets may overwhelm the system, leading to reduced early cortical responses (P1). This aligns with evidence from studies showing reduced P1 amplitude in response to simple sounds in ASD, as well as impaired modulation of P1 with changing stimulation rate and reduced habituation in ASD [40,41]. By 60 ms, the energy accumulation is already slower, and processing should become less demanding. However, this is where children with ASD showed the most pronounced deviation in N2 latency and P1N2 amplitude that might suggest a compensatory nature of the effect. Thus, the difficulty in ASD is not simply a matter of handling high-energy onsets, but rather a problem in adjusting processing dynamics when the acoustic input changes. The atypical P1N2 trajectory in ASD—decreasing at 60 ms but then tendency to rise at longer RTs, unlike the continued decrease in TD—further points to a disruption in the expected pattern of modulation across the RT continuum. This interpretation aligns with the hypothesis of atypical temporal envelope processing in ASD [11] and with reports that such difficulties are already detectable at the level of simple tone perception [40,42]. Importantly, auditory abilities in ASD are characterised by considerable individual variability [42,43], and different auditory parameters may be processed by distinct neural mechanisms, with their disruption in ASD not being uniform across domains [42]. The present findings extend this view by showing that within a single temporal parameter—RT—the pattern of modulation across RT durations is also disrupted in a non-uniform manner, with the pronounced deviation occurring at the transition between rapid and gradual onsets.

## 5. Conclusions

This study provides the first evidence of atypical auditory RT processing in children with autism spectrum disorder compared to typically developing peers using a passive block design with a range of RTs from 15 to 240 ms.

The ASD group showed a qualitatively different profile of neural modulation: early sensory responses (P1) lacked the typical amplitude decrease with RT, and later processes (N2 latency, P1N2 amplitude) deviated from the typical progression specifically between 15 and 60 ms. This disruption was most pronounced at the transition from abrupt to gradual onsets (60 ms), where the expected differentiation of acoustic cues appeared altered. Notably, cortical responses to the most gradual onsets did not differ between groups, suggesting that the temporal envelope processing deficit in ASD is selective rather than global.

These findings provide electrophysiological evidence for atypical processing of auditory temporal-envelope cues in ASD, demonstrating that such differences can be detected at the level of cortical responses to simple non-speech sounds. More broadly, the results highlight the importance of considering the entire temporal range when investigating auditory processing in ASD, as atypicalities may not be uniform across RTs. Future research should adopt longitudinal designs to examine whether early delays in neural evaluation predict later outcomes in ASD.

## 6. Limitations

Several limitations of the present study should be acknowledged. First, the sample size (N = 42, with 21 children per group) is comparable to many studies in developmental neurophysiology, but it remains relatively modest. Thus, replication in larger cohorts is necessary to confirm the reliability of the findings, particularly for effects that did not survive FDR correction. Second, despite careful artifact rejection and adaptive peak detection procedures, the analysis of ERP components in pediatric populations is inherently challenging due to increased noise levels and inter-individual variability in waveform morphology. This should be considered when interpreting null findings. Third, the cross-sectional design precludes any conclusions about causality or developmental trajectories. While we observed age-related changes in N2 amplitude and specific differences in the ASD group, longitudinal studies are needed to determine whether early auditory processing differences predict later developmental outcomes and to characterize the maturational trajectories of these neural markers, which can be specific in groups with neurodevelopmental disorders. Fourth, the age range of 4–10 years represents a period of rapid auditory cortical development [15], and the relatively small sample per group may have reduced sensitivity to detecting age-related interactions or more subtle group differences. Although we included age as a covariate in our statistical models, future studies with larger samples and more narrowly defined age bands would be valuable to characterize the developmental trajectory of RT processing in ASD. Fifth, we acknowledge that the heterogeneity of the ASD sample, with children ranging from minimally verbal to fluent speech and with varying SRS-2 scores (including some below the clinical threshold), may have masked subgroup-specific effects. While all participants had confirmed ADOS-2 diagnoses, future studies with larger samples and language-matched designs are needed to clarify whether the observed electrophysiological patterns correlate with specific clinical features such as language ability or social-communication severity, and whether longer RT processing is differentially affected in verbally fluent versus minimally verbal children with ASD. Sixth, peripheral hearing was confirmed through a parental report rather than a standardized audiometric assessment. While children with known or suspected hearing impairment were excluded, subclinical hearing differences cannot be fully ruled out. In addition, objective measures of language ability and non-verbal cognitive level (PLS-5 and Leiter-3) were available only for a subset of participants and were therefore not included in the main statistical models. Future studies should include standardized audiological screening and comprehensive language and cognitive assessments to ensure that auditory processing differences observed in ASD are not confounded by peripheral hearing status, language ability, or cognitive level.

## Figures and Tables

**Figure 1:**
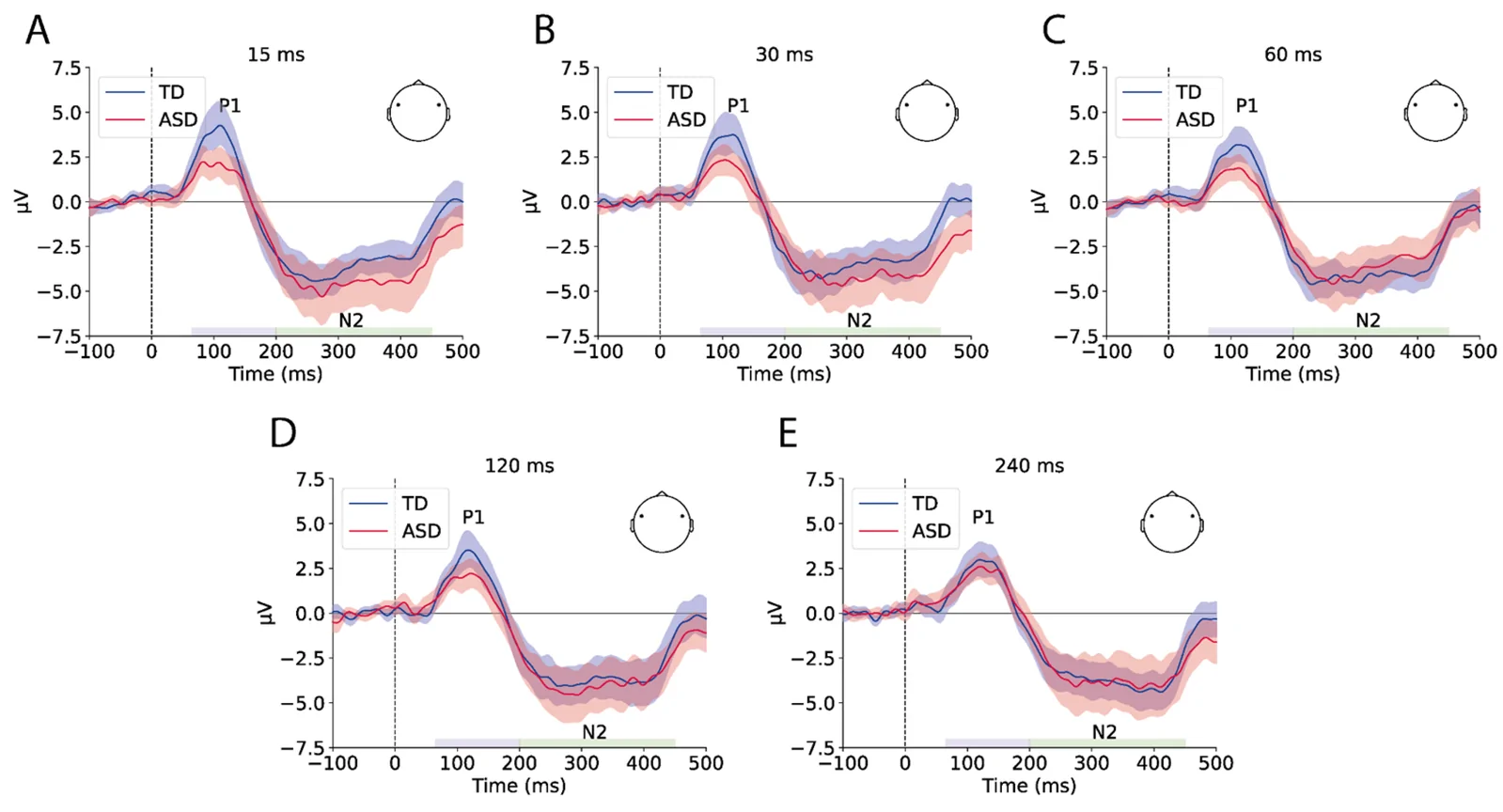
Event-related potentials (ERPs) on averaged FC5 and FC6 channels for the typically developing (TD, blue line) and autism spectrum disorder (ASD, red line) groups across rise time (RT) conditions: (**A**) 15 ms, (**B**) 30 ms, (**C**) 60 ms, (**D**) 120 ms, (**E**) 240 ms. Shading corresponds to the 95% confidence level. Bars over the x-axis indicate the time windows in which peaks were detected (violet—P1, green—N2).

**Figure 2:**
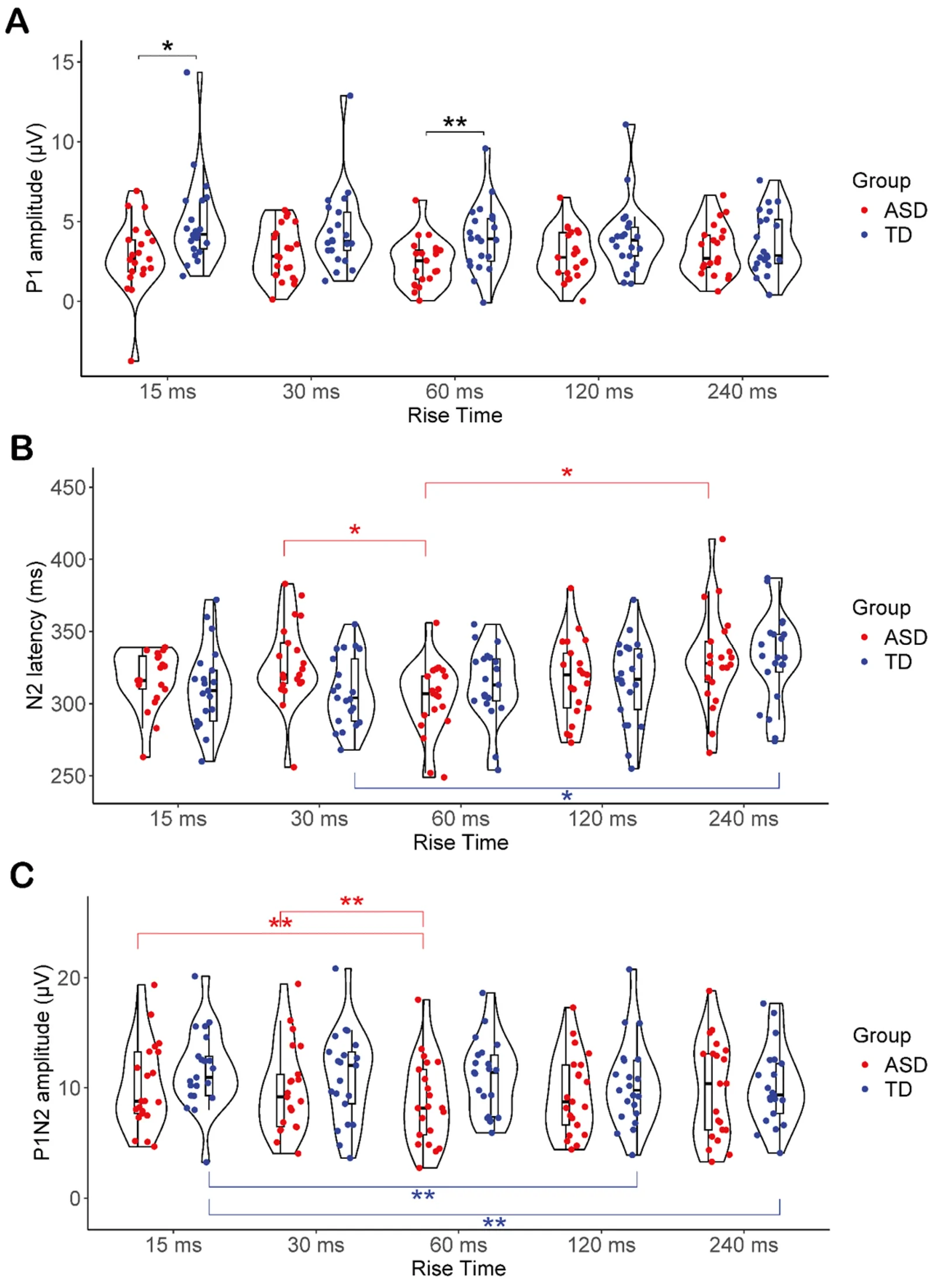
Group differences in P1 amplitude, N2 latency, and P1N2 amplitude across RTs (15–240 ms). (**A**) Reduced P1 amplitude in ASD compared to TD at 15 and 60 ms RT conditions (trend at 30 ms). (**B**) Different patterns of N2 latency change in the ASD and TD groups. (**C**) Different patterns of P1N2 amplitude change in the ASD and TD groups. Asterisks denote the level of statistical significance (* *p* < 0.05, ** *p* < 0.01, FDR-corrected).

**Table 1:** Group comparisons (ASD vs. TD) for P1 amplitude.

RT (ms)	ASD Mean (µV)	TD Mean (µV)	t	df	*p* (Raw)	*p* (FDR)
15	2.83	4.92	−2.68	40	0.011	0.026
30	3.04	4.40	−2.09	40	0.043	0.072
60	2.45	3.98	−2.73	40	0.010	0.026
120	2.92	3.96	−1.74	40	0.089	0.111
240	3.22	3.68	−0.85	40	0.400	0.400

**Table 2:** Group comparisons (ASD vs. TD) for N2 latency.

RT (ms)	ASD Mean (ms)	TD Mean (ms)	t	df	*p* (Raw)	*p* (FDR)
15	317.10	311.00	0.86	40	0.394	0.657
30	327.90	308.00	2.40	40	0.021	0.106
60	303.86	313.95	−1.25	40	0.217	0.543
120	317.05	315.90	0.14	40	0.889	0.931
240	330.24	330.95	−0.09	40	0.931	0.931

**Table 3:** Group comparisons (ASD vs. TD) for P1N2 amplitude.

RT (ms)	ASD Mean (µV)	TD Mean (µV)	t	df	*p* (Raw)	*p* (FDR)
15	10.12	11.60	−1.28	40	0.208	0.521
30	9.84	11.00	−0.93	40	0.359	0.551
60	8.74	11.00	−2.00	40	0.052	0.262
120	9.43	10.30	−0.78	40	0.441	0.551
240	9.78	10.10	−0.22	40	0.824	0.824

## Data Availability

The EEG data supporting the findings of this study are available in the Open Science Framework (OSF) repository at https://osf.io/ycfvr/overview (accessed on 3 July 2026). The data are publicly accessible under a Creative Commons Attribution 4.0 International (CC-BY 4.0) license.

## Supplementary Materials

The following supporting information can be downloaded at: https://www.mdpi.com/article/10.3390/brainsci16101010/s1. Figure S1: P1 topography for all rise times in ASD and TD groups; Figure S2: N2 topography for all rise times in ASD and TD groups; Table S1: Demographic characteristics and SRS-2 scores for age- and sex-matched pairs of children with ASD and TD peers; Table S2: Demographic, language, and cognitive characteristics of the sample Table S3: Linear mixed model results for P1 amplitude; Table S4: Linear mixed model results for P1 latency; Table S5: Linear mixed model results for N2 latency; Table S6: Linear mixed model results for N2 amplitude; Table S7: Linear mixed model results for P1N2 amplitude; Table S8: Pairwise comparisons of P1 amplitude across rise time levels (whole sample); Table S9: Pairwise comparisons of P1 amplitude across rise time levels within the ASD group; Table S10: Pairwise comparisons of P1 amplitude across rise time levels within the TD group; Table S11: Pairwise comparisons of P1 latency across rise time levels (whole sample); Table S12: Pairwise comparisons of N2 latency across rise time levels (whole sample); Table S13: Pairwise comparisons of N2 latency across rise time levels within the ASD group; Table S14: Pairwise comparisons of N2 latency across rise time levels within the TD group; Table S15: Pairwise comparisons of P1N2 amplitude across rise time levels (whole sample); Table S16: Pairwise comparisons of P1N2 amplitude across rise time levels within the ASD group; Table S17: Pairwise comparisons of P1N2 amplitude across rise time levels within the TD group.

## Abbreviations

The following abbreviations are used in this manuscript:

RT	Rise time
ERP	Event-related potential
EEG	Electroencephalography
ASD	Autism spectrum disorder
TD	Typically developing
MMN	Mismatch negativity

## References

[1] Kalashnikova M., Goswami U., Burnham D. (2019). Sensitivity to Amplitude Envelope Rise Time in Infancy and Vocabulary Development at 3 Years: A Significant Relationship. Dev. Sci..

[2] Phillips D.P., Hall S.E., Boehnke S.E. (2002). Central Auditory Onset Responses, and Temporal Asymmetries in Auditory Perception. Hear. Res..

[3] Walsh M.A., Diehl R.L. (1991). Formant Transition Duration and Amplitude Rise Time as Cues to the Stop/Glide Distinction. Q. J. Exp. Psychol. Sect. A.

[4] Howell P., Rosen S. (1983). Production and Perception of Rise Time in the Voiceless Affricate/Fricative Distinction. J. Acoust. Soc. Am..

[5] Leong V., Hämäläinen J., Soltész F., Goswami U. (2011). Rise Time Perception and Detection of Syllable Stress in Adults with Developmental Dyslexia. J. Mem. Lang..

[6] Leong V., Goswami U. (2014). Assessment of Rhythmic Entrainment at Multiple Timescales in Dyslexia: Evidence for Disruption to Syllable Timing. Hear. Res..

[7] Manasevich V., Kostanian D., Rogachev A., Sysoeva O. (2026). EEG Correlates of Auditory Rise Time Processing: A Systematic Review. bioRxiv.

[8] Stefanics G., Fosker T., Huss M., Mead N., Szucs D., Goswami U. (2011). Auditory Sensory Deficits in Developmental Dyslexia: A Longitudinal ERP Study. Neuroimage.

[9] Hämäläinen J.A., Leppänen P.H.T., Guttorm T.K., Lyytinen H. (2008). Event-Related Potentials to Pitch and Rise Time Change in Children with Reading Disabilities and Typically Reading Children. Clin. Neurophysiol..

[10] Peter V., Kalashnikova M., Burnhammarcs D. (2016). Neural Processing of Amplitude and Formant Rise Time in Dyslexia. Dev. Cogn. Neurosci..

[11] Alcántara J.I., Cope T.E., Cope W., Weisblatt E.J. (2012). Auditory Temporal-Envelope Processing in High-Functioning Children with Autism Spectrum Disorder. Neuropsychologia.

[12] Dynia J.M., Bean A., Justice L.M., Kaderavek J.N. (2019). Phonological Awareness Emergence in Preschool Children with Autism Spectrum Disorder. Autism Dev. Lang. Impair..

[13] Molfese D.L., Molfese V.J. (1988). Right-Hemisphere Responses from Preschool Children to Temporal Cues to Speech and Nonspeech Materials: Electrophysiological Correlates. Brain Lang..

[14] De Sanctis P. (2009). Right Hemispheric Contributions to Fine Auditory Temporal Discriminations: High-Density Electrical Mapping of the Duration Mismatch Negativity (MMN). Front. Integr. Neurosci..

[15] Bishop D.V.M., Anderson M., Reid C., Fox A.M. (2011). Auditory Development between 7 and 11 Years: An Event-Related Potential (ERP) Study. PLoS ONE.

[16] Lord C., Rutter M., DiLavore P.C., Risi S., Gotham K., Bishop S. (2012). Autism Diagnostic Observation Schedule, Second Edition (ADOS-2).

[17] Rutter M., Le Couteur A., Lord C. (2003). Autism Diagnostic Interview-Revised (ADI-R).

[18] Falkmer T., Anderson K., Falkmer M., Horlin C. (2013). Diagnostic Procedures in Autism Spectrum Disorders: A Systematic Literature Review. Eur. Child. Adolesc. Psychiatry.

[19] Constantino J.N., Gruber C.P. (2012). Social Responsiveness Scale, Second Edition (SRS-2).

[20] Constantino J.N., Davis S.A., Todd R.D., Schindler M.K., Gross M.M., Brophy S.L., Metzger L.M., Shoushtari C.S., Splinter R., Reich W. (2003). Validation of a brief quantitative measure of autistic traits: Comparison of the social responsiveness scale with the autism diagnostic interview-revised. J. Autism Dev. Disord..

[21] Talantseva O.I., An I.O., Zhukova M.A., Trubitsyna A.N., Teedemaa A.V., Grigorenko E.L. (2022). Psychometric Properties of the Preschool Language Scales, Fifth Edition (PLS-5) in Russian-Speaking Children. Clin. Psychol. Spec. Educ..

[22] Roid G.H., Miller L.J., Pomplun M., Koch C., Sorokin A. (2014). Leiter International Performance Scale, Third Edition (Leiter-3): Russian Adaptation.

[23] Picton T.W., Bentin S., Berg P., Donchin E., Hillyard S.A., Johnson R., Miller G.A., Ritter W., Ruchkin D.S., Rugg M.D. (2000). Guidelines for Using Human Event-related Potentials to Study Cognition: Recording Standards and Publication Criteria. Psychophysiology.

[24] Luck S.J. (2014). An Introduction to the Event-Related Potential Technique.

[25] Čeponiene R., Rinne T., Näätänen R. (2002). Maturation of Cortical Sound Processing as Indexed by Event-Related Potentials. Clin. Neurophysiol..

[26] Ponton C.W., Eggermont J.J., Kwong B., Don M. (2000). Maturation of Human Central Auditory System Activity: Evidence from Multi-Channel Evoked Potentials. Clin. Neurophysiol..

[27] Bates D., Mächler M., Bolker B., Walker S. (2015). Fitting Linear Mixed-Effects Models Using Lme4. J. Stat. Soft..

[28] Kuznetsova A., Brockhoff P.B., Christensen R.H.B. (2017). lmerTest Package: Tests in Linear Mixed Effects Models. J. Stat. Soft..

[29] Plakas A., van Zuijen T., van Leeuwen T., Thomson J., van der Leij A. (2013). Impaired Non-Speech Auditory Processing at a Pre-Reading Age Is a Risk-Factor for Dyslexia but Not a Predictor: An ERP Study. Cortex.

[30] Wunderlich J.L., Cone-Wesson B.K. (2006). Maturation of CAEP in Infants and Children: A Review. Hear. Res..

[31] Navarrete-Arroyo S., Putkinen V., Kujala T., Virtala P. (2025). Maturation of Speech-Elicited Event-Related Potentials from Birth to 28 Months: Typical Development with Minor Effects of Dyslexia Risk. Biol. Psychol..

[32] Sharma A., Glick H., Campbell J., Biever A. (2013). Central Auditory Development in Children with Hearing Impairment: Clinical Relevance of the P1 CAEP Biomarker in Children with Multiple Disabilities. Hear. Balance Commun..

[33] Godey B., Schwartz D., De Graaf J.B., Chauvel P., Liégeois-Chauvel C. (2001). Neuromagnetic Source Localization of Auditory Evoked Fields and Intracerebral Evoked Potentials: A Comparison of Data in the Same Patients. Clin. Neurophysiol..

[34] Butler B.E., Trainor L.J. (2012). Sequencing the Cortical Processing of Pitch-Evoking Stimuli Using EEG Analysis and Source Estimation. Front. Psychol..

[35] Zoellner S., Benner J., Zeidler B., Seither-Preisler A., Christiner M., Seitz A., Goebel R., Heinecke A., Wengenroth M., Blatow M. (2019). Reduced Cortical Thickness in Heschl’s Gyrus as an in Vivo Marker for Human Primary Auditory Cortex. Hum. Brain Mapp..

[36] Skinner P.H., Jones H.C. (1968). Effects of Signal Duration and Rise Time on the Auditory Evoked Potential. J. Speech Hear. Res..

[37] Näätänen R., Paavilainen P., Rinne T., Alho K. (2007). The Mismatch Negativity (MMN) in Basic Research of Central Auditory Processing: A Review. Clin. Neurophysiol..

[38] Folstein J.R., Van Petten C. (2008). Influence of Cognitive Control and Mismatch on the N2 Component of the ERP: A Review. Psychophysiology.

[39] Hakvoort B., Van Der Leij A., Maurits N., Maassen B., Van Zuijen T.L. (2015). Basic Auditory Processing Is Related to Familial Risk, Not to Reading Fluency: An ERP Study. Cortex.

[40] Rotschafer S.E. (2021). Auditory Discrimination in Autism Spectrum Disorder. Front. Neurosci..

[41] Gonçalves A.M., Monteiro P. (2023). Autism Spectrum Disorder and Auditory Sensory Alterations: A Systematic Review on the Integrity of Cognitive and Neuronal Functions Related to Auditory Processing. J. Neural Transm..

[42] Kargas N., López B., Reddy V., Morris P. (2015). The Relationship Between Auditory Processing and Restricted, Repetitive Behaviors in Adults with Autism Spectrum Disorders. J. Autism Dev. Disord..

[43] O’Connor K. (2012). Auditory Processing in Autism Spectrum Disorder: A Review. Neurosci. Biobehav. Rev..

