# Supplementary material for: Neurophysiological Processing of Rise Time in Children with Autism Spectrum Disorder
Source: Brain Sci. 2026 Sep 23;16(10):1010. doi: 10.3390/brainsci16101010 (PMC13702687; doi:10.3390/brainsci16101010)
Supplement: Supplementary file 1 [file brainsci-16-01010-s001.zip › brainsci-4512821-supplementary.pdf]

Supplementary Figure S1. P1 topography for all rise times, ASD and TD groups

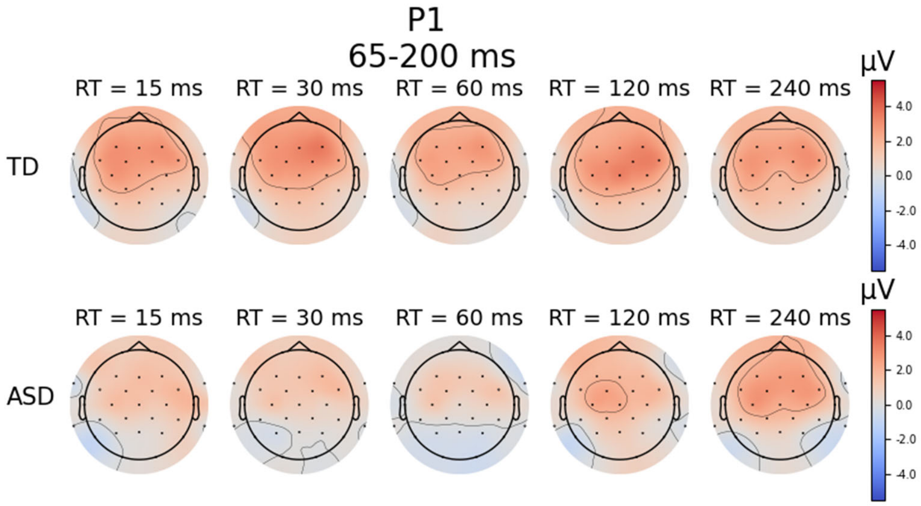

Supplementary Figure S2. N2 topography for all rise times, ASD and TD groups

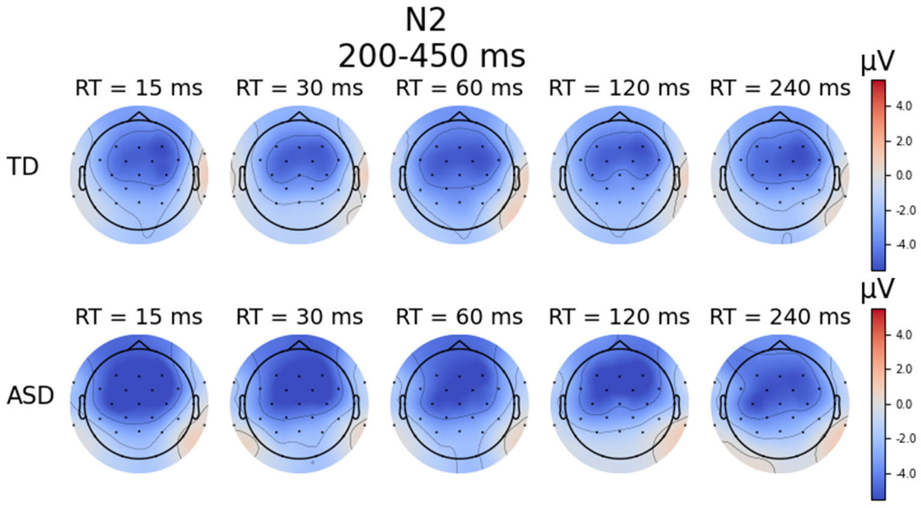

Table S1. Demographic characteristics and SRS-2 scores for age- and sex-matched pairs of children with autism spectrum disorder (ASD) and typically developing (TD) peers

ID TD	Age TD	Sex TD	SRS raw scores TD	SRS T-scores TD	ID ASD	Age ASD	Sex ASD	SRS raw scores ASD	SRS T-scores ASD
NP102203	52	f	65	59	NP062515	52	f	58	56
NP042317	56	f	25	44	NP062518	57	f	100	72
NP112221	61	m	27	44	NP112216	61	m	89	68
NP112301	61	m	21	42	NP062522	61	m	109	76
NP112311	63	m	30	45	NP112202	63	m	73	62

NP112313	65	m	36	48	NP062520	65	m	106	75
NP112309	73	f	41	50	NP062513	74	f	51	54
NP042324	80	m	27	44	NP022312	80	m	67	60
NP112225	81	m	62	58	NP042323	81	m	78	64
NP112320_2	84	m	21	45	NP042316	83	m	70	61
NP032401	86	m	36	48	NP062502	89	m	110	80
NP122202	90	m	29	48	NP062506	91	m	86	71
NP042326_2	91	m	21	45	NP062508	92	m	31	46

NP052305	93	m	32	49	NP062503	93	m	69	64
NP112302	94	m	21	45	NP042330	94	m	101	77
NP042315	96	f	33	52	NP042322	95	f	91	76
NP112217_2	97	m	29	48	NP062501	94	m	137	90
NP052401	97	m	43	54	NP062523	99	m	84	70
NP102311	106	f	61	57	NP062514	103	f	94	77
NP102302	112	f	23	47	NP062510	107	f	63	64
NP052314	122	m	46	55	NP062509	119	m	65	62

Table S2. Demographic, language, and cognitive characteristics of the sample.

Measure	ASD (n = 21)	TD (n = 21)
Age, months, M (SD)	83.5 (18.2)	83.8 (19.1)
Sex, n female	6	6
PLS-5 Receptive, SS, M (SD)	63.7 (19.8), n = 16	96.8 (18.8), n = 14
PLS-5 Expressive, SS, M (SD)	59.4 (18.9), n = 16	95.1 (23.9), n = 14
PLS-5 Total Language, SS, M (SD)	60.6 (19.1), n = 16	95.8 (21.1), n = 14
Leiter-3 Non-verbal IQ, M (SD)	93.0 (12.6), n = 18	107.1 (11.7), n = 18
Leiter-3 Non-verbal Memory, M (SD)	91.1 (26.1), n = 14	103.6 (17.6), n = 18

Note. PLS-5 = Preschool Language Scale, 5th edition; SS = standard score. Leiter-3 = Leiter International Performance Scale, 3rd edition. n indicates the number of participants for whom each measure was available.For Leiter-3 Non-verbal Memory, four children in the ASD group did not complete the task due to their functional state. Group comparisons were not performed due to incomplete data coverage across measures.

Table S3. Linear mixed model results for P1 amplitude

Effect	Num DF	Den DF	F	p	η^2p
**Group**	**1**	**42**	**6.63**	**.014**	**.136**
**RT**	**4**	**378**	**2.42**	**.048**	**.025**
**Electrode**	**1**	**378**	**7.28**	**.007**	**.019**
Age	1	42	1.18	.284	.027
**Group × RT**	**4**	**378**	**3.29**	**.011**	**.034**
Group × Electrode	1	378	0.04	.851	.000
RT × Electrode	4	378	0.12	.974	.001
Group × RT × Electrode	4	378	0.61	.659	.006

Table S4. Linear mixed model results for P1 latency

Effect	Num DF	Den DF	F	p	$\eta^2 p$
Group	1	42	0.07	.799	.002
**RT**	**4**	**378**	**24.79**	**< .001**	**.208**
**Electrode**	**1**	**378**	**6.30**	**.013**	**.016**
Age	1	42	2.66	.110	.060
Group × RT	4	378	1.71	.147	.018
Group × Electrode	1	378	2.68	.103	.007
RT × Electrode	4	378	1.08	.369	.011
Group × RT × Electrode	4	378	0.45	.774	.005

Table S5. Linear mixed model results for N2 latency

Effect	Num DF	Den DF	F	p	$\eta^2 p$
Group	1	42	0.35	.559	.008
**RT**	**4**	**378**	**7.26**	**< .001**	**.071**
Electrode	1	378	3.21	.074	.008
Age	1	42	0.21	.649	.005
**Group × RT**	**4**	**378**	**3.14**	**.015**	**.032**
Group × Electrode	1	378	0.09	.770	.000
**RT × Electrode**	**4**	**378**	**0.33**	**.857**	**.003**
Group × RT × Electrode	4	378	0.42	.797	.004

Table S6. Linear mixed model results for N2 amplitude

Effect	Num DF	Den DF	F	p	η^2p
Group	1	42	0.02	.894	.000
RT	4	378	1.08	.366	.011
**Electrode**	**1**	**378**	**5.26**	**.022**	**.014**
**Age**	**1**	**42**	**6.52**	**.014**	**.134**
Group × RT	4	378	1.19	.316	.012
Group × Electrode	1	378	0.47	.493	.001
RT × Electrode	4	378	0.38	.823	.004
Group × RT × Electrode	4	378	0.23	.923	.002

Table S7. Linear mixed model results for P1N2 amplitude

Effect	Num DF	Den DF	F	p	η^2p
Group	1	42	1.29	.263	.030
**RT**	**4**	**378**	**4.23**	**.002**	**.043**
**Electrode**	**1**	**378**	**23.31**	**< .001**	**.058**
**Age**	**1**	**42**	**4.67**	**.036**	**.100**
**Group × RT**	**4**	**378**	**2.74**	**.029**	**.028**
Group × Electrode	1	378	0.94	.333	.002
RT × Electrode	4	378	0.57	.687	.006
Group × RT × Electrode	4	378	0.43	.785	.005

Table S8. P1 amplitude, pairwise RT comparisons, all sample

RT1	RT2	t	df	p (raw)	p (FDR)
15ms	30ms	0.481	41	.633	.703
15ms	60ms	2.087	41	.043	.249
15ms	120ms	1.373	41	.177	.448
15ms	240ms	1.185	41	.243	.448
30ms	60ms	2.022	41	.050	.249
30ms	120ms	1.053	41	.298	.448
30ms	240ms	0.991	41	.327	.448
60ms	120ms	-1.089	41	.283	.448
60ms	240ms	-0.929	41	.358	.448
120ms	240ms	-0.024	41	.981	.981

Note. *Paired t-tests. FDR correction (Benjamini-Hochberg) was applied to 10 comparisons.*

Table S9. Pairwise comparisons of P1 amplitude across rise time levels within ASD group (paired t-tests)

RT1	RT2	t	df	p (raw)	p (FDR)
15ms	30ms	-0.525	20	.605	.757
15ms	60ms	0.797	20	.435	.725
15ms	120ms	-0.209	20	.837	.837
15ms	240ms	-0.965	20	.346	.692
30ms	60ms	1.764	20	.093	.324
30ms	120ms	0.347	20	.732	.813
30ms	240ms	-0.606	20	.552	.757
60ms	120ms	-1.740	20	.097	.324

60ms	240ms	-2.756	20	.012	.122
120ms	240ms	-1.333	20	.197	.494

Note. No comparisons survived FDR correction (all p(FDR) > .05).

Table S10. Pairwise comparisons of P1 amplitude across rise time levels within TD group (paired t-tests)

RT1	RT2	t	df	p (raw)	p (FDR)
15ms	30ms	1.084	20	.291	.426
15ms	60ms	2.260	20	.035	.117
15ms	120ms	2.304	20	.032	.117
15ms	240ms	2.263	20	.035	.117
30ms	60ms	1.107	20	.281	.426
30ms	120ms	1.068	20	.298	.426

30ms	240ms	1.582	20	.129	.324
60ms	120ms	0.079	20	.938	.938
60ms	240ms	0.791	20	.438	.548
120ms	240ms	0.546	20	.591	.657

Note. No comparisons survived FDR correction (all p(FDR) > .05).

Table S11. Pairwise comparisons of P1 latency across rise time levels (whole sample, averaged across electrodes)

RT1	RT2	t	df	p (raw)	p (FDR)
15ms	30ms	0.526	41	.601	.601
15ms	60ms	-0.642	41	.525	.583
**15ms**	**120ms**	**-3.716**	**41**	**< .001**	**.001**
**15ms**	**240ms**	**-5.934**	**41**	**< .001**	**< .001**

30ms	60ms	-1.339	41	.188	.235
**30ms**	**120ms**	**-4.706**	**41**	**< .001**	**< .001**
**30ms**	**240ms**	**-7.807**	**41**	**< .001**	**< .001**
**60ms**	**120ms**	**-2.731**	**41**	**.009**	**.013**
**60ms**	**240ms**	**-6.010**	**41**	**< .001**	**< .001**
**120ms**	**240ms**	**-3.064**	**41**	**.004**	**.006**

Note. Paired t-tests. FDR correction (Benjamini-Hochberg) was applied to 10 comparisons. Significant p(FDR) < .05 are in bold.

Table S12. Pairwise comparisons of N2 latency across rise time levels (whole sample, averaged across electrodes, paired t-tests)

RT1	RT2	t	df	p (raw)	p (FDR)
15ms	30ms	-1.086	41	.284	.355
15ms	60ms	1.244	41	.221	.315

15ms	120ms	-0.558	41	.580	.644
**15ms**	**240ms**	**-3.156**	**41**	**.003**	**.015**
30ms	60ms	1.872	41	.068	.137
30ms	120ms	0.362	41	.719	.719
30ms	240ms	-2.398	41	.021	.053
60ms	120ms	-1.512	41	.138	.230
**60ms**	**240ms**	**-3.808**	**41**	**< .001**	**.005**
**120ms**	**240ms**	**-2.731**	**41**	**.009**	**.031**

Note. Paired t-tests. FDR correction (Benjamini-Hochberg) was applied to 10 comparisons. Significant p(FDR) < .05 are in bold.

Table S13. Pairwise comparisons of N2 latency across rise time levels within ASD group (paired t-tests)

RT1	RT2	t	df	p (raw)	p (FDR)
15ms	30ms	-1.940	20	.067	.135
15ms	60ms	2.229	20	.037	.125

15ms	120ms	0.009	20	.993	.993
15ms	240ms	-1.929	20	.068	.135
**30ms**	**60ms**	**3.259**	**20**	**.004**	**.022**
30ms	120ms	1.682	20	.108	.135
30ms	240ms	-0.311	20	.759	.843
60ms	120ms	-1.746	20	.096	.135
**60ms**	**240ms**	**-3.209**	**20**	**.004**	**.022**
120ms	240ms	-1.711	20	.103	.135

Note. Paired t-tests. FDR correction (Benjamini-Hochberg) was applied to 10 comparisons. Significant p(FDR) < .05 are in bold.

Table S14. Pairwise comparisons of N2 latency across rise time levels within TD group (paired t-tests)

RT1	RT2	t	df	p (raw)	p (FDR)
15ms	30ms	0.391	20	.700	.743

15ms	60ms	-0.540	20	.595	.743
15ms	120ms	-0.691	20	.497	.711
15ms	240ms	-2.473	20	.023	.113
30ms	60ms	-0.922	20	.368	.613
30ms	120ms	-1.069	20	.298	.595
**30ms**	**240ms**	**-3.395**	**20**	**.003**	**.029**
60ms	120ms	-0.332	20	.743	.743
60ms	240ms	-2.147	20	.044	.117
120ms	240ms	-2.121	20	.047	.117

Note. Paired t-tests. FDR correction (Benjamini-Hochberg) was applied to 10 comparisons. Significant p(FDR) < .05 are in bold.

Table S15. Pairwise comparisons of P1N2 amplitude across rise time levels (whole sample, averaged across electrodes)

RT1	RT2	t	df	p (raw)	p (FDR)
15ms	30ms	1.632	41	.110	.184
**15ms**	**60ms**	**3.617**	**41**	**< .001**	**.008**
**15ms**	**120ms**	**3.414**	**41**	**.001**	**.008**
**15ms**	**240ms**	**2.909**	**41**	**.006**	**.019**
30ms	60ms	2.330	41	.025	.062
30ms	120ms	2.035	41	.048	.097
30ms	240ms	1.453	41	.154	.220
60ms	120ms	-0.123	41	.903	.903
60ms	240ms	-0.227	41	.821	.903
120ms	240ms	-0.153	41	.880	.903

Table S16. Pairwise comparisons of P1N2 amplitude across rise time levels within ASD group

RT1	RT2	t	df	p (raw)	p (FDR)
15ms	30ms	1.062	20	.301	.501
**15ms**	**60ms**	**3.870**	**20**	**.001**	**.009**
15ms	120ms	1.563	20	.134	.268
15ms	240ms	0.714	20	.483	.537
**30ms**	**60ms**	**3.589**	**20**	**.002**	**.009**
30ms	120ms	0.939	20	.359	.513
30ms	240ms	0.120	20	.906	.906
60ms	120ms	-2.289	20	.033	.083
60ms	240ms	-2.563	20	.019	.062
120ms	240ms	-0.779	20	.445	.537

Table S17. Pairwise comparisons of P1N2 amplitude across rise time levels within TD group

RT1	RT2	t	df	p (raw)	p (FDR)
15ms	30ms	1.257	20	.223	.279
15ms	60ms	1.512	20	.146	.209
**15ms**	**120ms**	**3.490**	**20**	**.002**	**.012**
**15ms**	**240ms**	**3.809**	**20**	**.001**	**.011**
30ms	60ms	0.138	20	.891	.891
30ms	120ms	2.160	20	.043	.135
30ms	240ms	1.906	20	.071	.135
60ms	120ms	1.839	20	.081	.135
60ms	240ms	2.035	20	.055	.135
120ms	240ms	0.713	20	.484	.538
